# The rs2229611 (*G6PC:*c.*23 T>C) is associated with glycogen storage disease type Ia in Brazilian patients

**DOI:** 10.1016/j.ymgmr.2020.100659

**Published:** 2020-10-20

**Authors:** Franciele Cabral Pinheiro, Fernanda Sperb-Ludwig, Juliana Maria Fagundes Verch, Bruna Bento dos Santos, Carolina Fischinger Moura de Souza, Ida Vanessa Doederlein Schwartz

**Affiliations:** a Post Graduate Program in Genetics and Molecular Biology, Universidade Federal do Rio Grande do Sul (UFRGS), Porto Alegre, RS, Brazil; b Laboratory of Basic Research and Advanced Investigations in Neurosciences (BRAIN), Hospital de Clínicas de Porto Alegre (HCPA), Porto Alegre, RS, Brazil; c Universidade Federal do Pampa (UNIPAMPA), Itaqui, RS, Brazil; d Universidade Federal de Ciências da Saúde de Porto Alegre (UFCSPA), Porto Alegre, RS, Brazil; e Medical Genetics Service, Hospital de Clínicas de Porto Alegre (HCPA), Porto Alegre, RS, Brazil

**Keywords:** Glycogenosis, GSD Ia, Linkage-disequilibrium, Inborn error of metabolism, Genetic biomarker

## Abstract

The rs2229611 SNP (*G6PC*:c.*23T>C) in the 3’UTR region of the *G6PC* gene affects the stability of the glucose-6-phosphatase mRNA and occurs in a higher frequency in patients with glycogenosis Ia (GSD Ia) in some populations. Herein, a group of Brazilian patients (*n* = 116) was analyzed by NGS and the frequency of rs2229611:T>C was determined. The linkage disequilibrium (LD) between pathogenic variants and the rs2229611:T>C SNP was evaluated. The results showed that the rs2229611:T>C is associated to GSD Ia and is in LD with the most frequent pathogenic variants in Brazilian patients with GSD Ia.

## 1 Introduction

Glycogenoses (GSDs) consist of a group of inborn errors of metabolism that affect the synthesis or degradation of glycogen. The most frequent and severe is GSD type I, an autosomal recessive disease that affects the glucose-6-phosphatase (G6Pase) complex. Pathogenic variants in *G6PC* and *SLC37A4* genes result in GSD Ia (OMIM 232200) and Ib (OMIM 232220), respectively [1].

To date, 128 pathogenic variants in *G6PC* have been described in Human Gene Mutation Database (HGMD, http://www.hgmd.cf.ac.uk/ac/gene.php?gene=G6PC) [2]. The genotype-phenotype relationship has been narrowed down to a small number of pathogenic variants; for instance, homozygosity for the NM_000151.4:c.648G > T (NP_00142.1:p.?), a splice site variant, is associated with an increased risk of hepatocellular carcinoma [3]. Studies have indicated that GSD Ia phenotypes are influenced by genetic and environmental modifying factors [4]. In this regard, the rs2229611 SNP (NG_011808.1:g.15652 T>C), located at the 3’ UTR of *G6PC* gene, has been shown to be a potential modulating factor for the severity of this disease. Karthi et al. (2017), demonstrated that the *G6PC:*c.*23C allele results in a shorter half-life mRNA than those resulting from the *G6PC:*c.*23T allele, also altering the spectrum of regulatory proteins that bind to the *G6PC* 3’ UTR region [5]. In addition, the rs2229611:T>C SNP frequency appears to be higher in patients with GSD Ia than in healthy controls [[5], [6], [7], [8], [9]]. The control sample studied by Lam et al. (1998) (*n* = 194) [6] was compared to 34 patients with GSD Ia by Wong et al. [7], showing that this SNP is in linkage disequilibrium (LD) with *G6PC* pathogenic variants, e.g., NM_000151.4:c.247C>T (p.Arg83Cys), NM_000151.4:c.248G>A (p.(Arg83His)) and c.648G>T (p.?) [7].

In order to evaluate the possible effect of the rs2229611 SNP in Brazilian patients with GSD Ia, we studied a cohort of 116 patients with hepatic GSD whose genotype had been previously described by Sperb-Ludwig (2019) [10]. So, we determined whether the frequency of the rs2229611:T > C SNP differs among GSD types and whether this SNP is associated with an earlier onset of symptoms in GSD Ia.

## 2 Material and methods

The patient genotype for the rs2229611:T>C SNP was determined by bioinformatics analysis of next-generation sequencing (NGS) results using Enlis Genomic (https://www.enlis.com/index.html) and Ion Reporter software using the same dataset of Sperb-Ludwig (2019) [10], and the frequency was compared to the frequency found in the gnomAD (v.3) database [11]. The sample consisted of 116 GSD patients (Ia = 50) (Table 1). The variants detected in *G6PC* were classified according ACMG (American College of Medical Genetics and Genomics) criteria [12]. The allele distribution among patients with different GSDs was analyzed via a likelihood-ratio χ2 test with residual adjustment. Residual values higher than two and *p*-values <0.05 were considered significantly different. One allele was discounted from the analyses because of the reported consanguinity among the parents of one patient, so the total number of alleles was 231.

**Table 1:** Frequency of the rs2229611 SNP (NM_000151.4:c.*23 T > C) in Brazilian patients with hepatic glycogen storage disease.

GSD type	Patients (n)	Alleles (n)	*G6PC:*c.*23 T n (%)	*G6PC:*c.*23C n (%)	*G6PC*:c.*23 TT n (%)	*G6PC*:c.*23 TC n (%)	*G6PC*:c.*23CC n (%)
Ia	50^#^	99	7 (7.1)	92 (92.9)*	1(2.0)	5 (10.0)	44 (88.0)
Others:	66	132	44 (33.3)	88 (66.7)	7 (10.6)	30 (45.5)	29 (43.9)
-Ib	23	46	10 (21.7)	36 (78.3)	ND	10 (43.5)	13 (56.5)
-III	13	26	9 (34.6)	17 (65.4)	1 (7.7)	7 (53.8)	5 (38.5)
-VI	2	4	1 (25.0)	3 (75.0)	ND	1 (50.0)	1 (50.0)
-IXα	16	32	13 (40.6)	19 (59.4)	4 (25.0)	5 (31.3)	7 (43.7)
-IXβ	6	12	6 (50.0)	6 (50.0)	1 (16.7)	4 (66.7)	1 (16.7)
-IXγ	6	12	5 (41.7)	7 (58.3)	1 (16.7)	3 (50.0)	2 (33.3)
Total	116	231	51 (22.1)	180 (77.9)	8 (6.9)	35 (30.2)	73 (62.9)

Note: The data are presented as number of alleles and the frequency in parenthesis. GSD = glycogen storage disease; ND = not detected; ^#^One patient had consanguineous parents, so one allele was discounted from the analyses. *Indicates the association of rs2229611 with GSD Ia (adjusted residual 4.8; *p* < 0.05).

To evaluate the LD between the *G6PC* gene variants and the rs2229611:T>C SNP, only variants in *G6PC* gene present in more than five alleles among patients with GSD Ia were considered. Patients with other GSD types (*n* = 132 alleles) were used as controls. The analyses were performed in the Haploview 4.2 software [13] and the D', r^2^ and LOD parameters were considered to determine the significance of the LD analysis. The parameter D' was used to characterizes how much two alleles were associated non-randomly - D' = 1 indicates the total disequilibrium. This parameter is independent of the allele frequencies. The r^2^ parameter is equivalent to Pearson correlation coefficient which has a similar significance to D', but is influenced by allele frequencies [14]. The LOD (logarithm of odds) score was used to indicates the probability of a disease and a marker are cosegregating due to linkage disequilibrium or to chance [15]. In reason of the sample size, it was considerate LOD > 2 as indicative of the significant linkage disequilibrium.

Information regarding the age of onset of symptomatology was available for 38 patients with GSD Ia. The ages of onset of symptoms were converted into months and categorized among genotypes (*G6PC*:*23 TC or *G6PC*:*23CC). The comparison between means was performed using the Mann Whitney nonparametric test for independent samples. The statistical analysis was performed in SPSS 18.1 (SPSS Inc., Chicago, IL, USA).

## 3 Results

The results are described in Table 1, Table 2. The data showed that the frequency of the rs2229611:T>C SNP is higher in GSD Ia but not in other types of hepatic GSD (Table 1). In the gnomAD (v.3) database [11], the total rs2229611:T > C SNP frequency was 73.2% (104,736 in 143,096 alleles), with variations from 45.6% to 81.6% in East Asian and European (Finnish) populations, respectively. The variants present in more than five alleles were classified according ACMG criteria as likely pathogenic: NM_000151.4:c.247C>T (p.Arg83Cys; PS3, PM3, PP1, PP3, BP1), NM_000151.4:c.161A>C (p.Gln54Pro; PS3, PM2, PM3, BP1), NM_000151.4:c.563-3C>G (p.?; PM2, PM3, PP3, PP4) and as pathogenic NM_000151.4:c.1039C>T (p.Gln347Ter; PVS1, PP3, PS3, PM3). LD analysis revealed that the c.247C>T variant presented two different haplotypes, one in LD with the *G6PC*:c.*23C allele (Table 2). The c.563-3G>C pathogenic variant was also in LD with the *G6PC*:c.*23C allele in our patients. Among patients with GSD Ia, five were *G6PC*:c.*23 TC heterozygous, 44 were *G6PC*:c.*23CC homozygous, and one was *G6PC*:c.*23 TT homozygous (Table 1). The median age at symptom onset was 3 (IQR = 2.25–6) months in *G6PC*:c.*23 TC patients (*n* = 4) and 3 (IQR = 0–6) months in *G6PC*:c.*23CC patients (*n* = 34). There was no significant difference among the groups (*p*-value = 0.51).

**Table 2:** Parameters of linkage disequilibrium analysis of rs2229611 (NM_000151.4:c.*23 T > C) with common variants in Brazilian patients with glycogen storage disease Ia.

Variant	D'	r^2^	LOD^c^
c.161A>C (p.Gln54Pro)^a^	1	0.009	<2
c.247C>T (p.Arg83Cys)^a^	0.85	0.043	≥2
c.563-3C>G (splice site)^a^	1	0.025	≥2
c.1039C>T (p.Gln347Ter)^b^	1	0.019	<2

Note: The LD analysis was performed in Haploview 4.2 software.

a likely pathogenic

b pathogenic

c LOD values higher than two indicate that LD is significant, this score is influenced by sample size.

## 4 Discussion

This is the first study that evaluated the frequency of the rs2229611:T > C SNP in patients with different hepatic GSDs. The data showed that the rs2229611:T>C SNP frequency among hepatic GSDs, except GSD Ia, is similar to that observed in controls of the gnomAD database (73.19%). The frequency of this SNP in our group of Brazilian patients with GSD Ia is ~93%, the highest value observed in the patient populations studied so far [[5], [6], [7], [8], [9]]. Our data also showed that the frequency of the *G6PC*:c.*23C allele is different between patients with GSD Ia and with other types of GSD (66.7%).

The gnomAD database analysis reinforced the conclusion of previous studies that the frequency of rs2229611:T>C SNP varies (45–85%) among different populations [[5], [6], [7], [8], [9]]. In this sense, Lam et al. (1998) suggested that rs2229611:T>C SNP could be used as a marker in Chinese and Hispanic populations for both carrier screening and prenatal diagnosis of GSD Ia in families whose two pathogenic variants have not been identified [6]. Thus, based on the rs2229611:T>C SNP frequency in Asian populations in gnomAD (45.6%), the suggested approach may be a good alternative, but in populations such as Finns (81.6%), this method can generate many false positives. In our sample, the frequency of homozygotes *G6PC*:c.*23CC is higher in GSD Ia (88%) when compared to other types of GSD (43.9%). Thus, in the Brazilian population, homozygosity of *G6PC*:c*23CC may indicate the diagnosis of GSD Ia in symptomatic patients. Therefore, the high frequency of *G6PC:*c.*23C allele in control populations makes the rs2229611:T>C a sensitive but non-specific biomarker for the diagnosis of GSD Ia.

The higher frequency of the rs2229611:T>C SNP in patients with GSD Ia could be related to the LD between this SNP and pathogenic *G6PC* variants. The analyses showed that two variants are in LD with the rs2229611:T>C SNP in our group of patients (Table 2). The c.247C>T variant was present in two different haplotypes in Brazil (Table 2), one linked with the *G6PC:*c.*23T allele. The second haplotype was in LD with the *G6PC:*c.*23C allele, as showed in Caucasian, Hispanic and Chinese patients [7] and in another group of Brazilian patients [9]. Our data suggest two origins for this allele in the Brazilian population. The c.809G>T (p.(Gly270Val)) variant could be in LD with the *G6PC:*c.*23T allele, but the number of alleles in the sample is two, thus preventing appropriate analysis. However, the only patient who was *G6PC*:c.*23 TT is also homozygous for the c.809G>T variant, reinforcing this hypothesis. Thirteen pathogenic variants that appear to be in LD with the rs2229611:T>C SNP (data not shown) were present in only one or two patients, thus hindering a more robust analysis. Thus, our data reinforce that this SNP is in LD with the most prevalent pathogenic variants in *G6PC* in the Brazilian patients.

The evidence obtained by Karthi and collaborators [5] suggests that the rs2229611:T>C SNP could be associated with greater GSD Ia severity; therefore, the age at symptom onset was analyzed. However, it was not possible to establish an association between this SNP and earlier GSD Ia onset in Brazilian patients. Due to the low frequency of the *G6PC:*c*23T allele in the Brazilian patients (7%, Table 1), there was insufficient statistical power to perform an association study.

The p.Arg83Cys have been previously evaluated by functional assays, and alone abolishes G6Pase activity [16]. So, the rs2229611:T>C SNP is not expected to increase the severity of GSD Ia when this variant is present. In the analysis of the SNP in constructs containing a 3’UTR portion of the *G6PC* gene associated with a luciferase expression vector, it was possible to observe the functional involvement of 3′UTR polymorphism rs2229611:T>C in the negative regulation of *G6PC* expression at mRNA levels, possibly by decreasing its stability [5]. Therefore, when this SNP is associated with pathogenic variants that have some residual activity, this may reflect in greater severity, as the mRNA instability can contribute to abolish the G6Pase activity.

Our data suggest the need to study a larger group of patients with GSD Ia who carry the *G6PC:*c.*23T allele to examine the association of the rs2229611:T>C SNP with the symptom severity, even though this is one of the largest cohorts ever analyzed for this purpose. Another approach would be in vitro expression studies with constructs carrying rs2229611-related variants to determine whether this SNP leads to altered expression levels. This approach would be valid for analyzing variants not yet studied or which are associated with a residual activity of G6Pase.

## 5 Conclusion

The data confirm that the rs2229611:T>C SNP is also associated to Brazilian patients with GSD Ia and showed the LD of this SNP with the c.247C>T and c.563-3C>G variants, the most frequent ones in this population.

## Declaration of Competing Interest

None.

## References

[1] Chen M.A., Weinstein D.A. (2016). Glycogen storage diseases: diagnosis, treatment and outcome. Transl. Sci. Rare Dis..

[2] Stenson P.D., Mort M., Edward V Ball ·., Evans K., Hayden M., Heywood S., Hussain M., Phillips A.D., Cooper D.N. (2017). The human gene mutation database: towards a comprehensive repository of inherited mutation data for medical research, genetic diagnosis and next-generation sequencing studies. Hum. Genet..

[3] Matern D., Seydewitz H.H., Bali D., Lang C., Chen Y.-T. (2002). Glycogen storage disease type I: diagnosis and phenotype/genotype correlation. Eur. J. Pediatr..

[4] Peeks F., Steunenberg T.A.H., De Boer F., Rubio-gozalbo M.E., Williams M., Burghard R., Rajas F., Oosterveer M.H., Weinstein D.A., Derks T.G.J. (2017). Clinical and biochemical heterogeneity between patients with glycogen storage disease type IA : the added value of CUSUM for metabolic control. J. Inherit. Metab. Dis..

[5] Karthi S., Rajeshwari M., Francis A., Saravanan M., Varalakshmi P., Houlden H., Thangaraj K., Ashokkumar B. (2017). 3′-UTR SNP rs2229611 in G6PC1 affects mRNA stability, expression and glycogen storage disease type-Ia risk. Clin. Chim. Acta..

[6] Lam C., Liang M., Pang C. (1998). Short report on DNA marker at candidate locus a novel Dral polymorphism in detection and prenatal diagnosis of glycogen storage disease t Y Pe l a. Clin. Genet..

[7] Wong L.-J.C., Liang M.-H., Hwu W.-L., Lam C.-W. (1998). Linkage disequilibrium and linkage analysis of the glucose-6-phosphatase gene. Hum. Genet..

[8] Kozak L., Francova H., Hrabincova E., Stastna S., Peskova K., Elleder M. (2000). Identification of mutations in the glucose-6-phosphatase gene in Czech and Slovak patients with glycogen storage disease type ia, including novel mutations K76N, V166A and 540del5. Hum. Mutat..

[9] Reis F. de C., Caldas H.C., Norato D.Y.J., Schwartz I.V.D., Giugliani R., Burin M.G., Sartorato E.L. (2001). Glycogen storage disease type Ia: molecular study in Brazilian patients. J. Hum. Genet..

[10] Sperb-ludwig F., Pinheiro F.C., Soares M.B., Nalin T., Ribeiro E.M., Steiner C.E., Valadares G., RibeiroPorta Eugênia, De Souza C.F.M., Schwartz I.V.D. (2019). Glycogen storage diseases : twenty - seven new variants in a cohort of 125 patients. Mol. Genet. Genomic Med..

[11] Karczewski K.J., Francioli L.C., Tiao G., Cummings B.B., Alföldi J., Wang Q., Collins R.L., Laricchia K.M., Ganna A., Birnbaum D.P., Gauthier L.D., Brand H., Solomonson M., Watts N.A., Rhodes D., Singer-Berk M., England E.M., Seaby E.G., Kosmicki J.A., Walters R.K., Tashman K., Farjoun Y., Banks E., Poterba T., Wang A., Seed C., Whiffin N., Chong J.X., Samocha K.E., Pierce-Hoffman E., Zappala Z., O A.H., Donnell-Luria E.V., Minikel B., Weisburd M., Lek J.S., Ware C., Vittal I.M., Armean L., Bergelson K., Cibulskis K.M., Connolly M., Covarrubias S., Donnelly S., Ferriera S., Gabriel J., Gentry N., Gupta T., Jeandet D., Kaplan C., Llanwarne R., Munshi S., Novod N., Petrillo D., Roazen V., Ruano-Rubio A., Saltzman M., Schleicher J., Soto K., Tibbetts C., Tolonen G., Wade M.E., Talkowski T.G.A.D., Consortium B.M., Neale M.J., Daly D.G. MacArthur (2019). Variation across 141,456 human exomes and genomes reveals the spectrum of loss-of-function intolerance across human protein-coding genes. BioRxiv.

[12] Richards S., Aziz N., Bale S., Bick D., Das S., Gastier-Foster J., Grody W.W., Hedge M., Lyon E., Spector E., Voelkerding K., Rehm H.L. (2015). Standards and guidelines for the interpretation of sequence variants: a joint consensus recommendation of the American College of Medical Genetics and Genomics and the Association for Molecular Pathology. Genet. Med..

[13] Barrett J.C., Fry B., Maller J., Daly M.J. (2005). Haploview: analysis and visualization of LD and haplotype maps. Bioinformatics..

[14] Slatkin M. (2008). Linkage disequilibrium - understanding the evolutionary past and mapping the medical future. Nat. Rev. Genet..

[15] Risch N. (1991). A note on multiple testing procedures in linkage analysis. Am. J. Hum. Genet..

[16] Shieh J.J., Terzioglu M., Hiraiwa H., Marsh J., Pan C.J., Chen L.Y., Chou J.Y. (2002). The molecular basis of glycogen storage disease type 1a. Structure and function analysis of mutations in glucose-6-phosphatase. J. Biol. Chem..

